# Cytochalasin H isolated from mangrove-derived endophytic fungus inhibits epithelial-mesenchymal transition and cancer stemness *via* YAP/TAZ signaling pathway in non-small cell lung cancer cells

**DOI:** 10.7150/jca.50512

**Published:** 2021-01-01

**Authors:** Zihan Xiu, Jiao Liu, Xin Wu, Xiangyong Li, Sanzhong Li, Xiaofeng Wu, Xiaohua Lv, Hua Ye, Xudong Tang

**Affiliations:** 1 Collaborative innovation center for antitumor active substance research and development, Institute of Biochemistry and Molecular Biology, Guangdong Medical University, Zhanjiang 524023, P.R. China.; 2 Guangdong Key Laboratory for Research and Development of Natural Drugs, Marine Medical Research Institute of Guangdong Zhanjiang, Department of Pharmacology, Guangdong Medical University, Zhanjiang 524023, P.R. China.; 3 Southern Marine Science and Engineering Guangdong Laboratory (Zhanjiang), Zhanjiang 524023, P.R. China.; 4 Guangdong Provincial Key Laboratory of Medical Molecular Diagnostics, Dongguan Key Laboratory of Medical Bioactive Molecular Developmental and Translational Research, Guangdong Medical University, Dongguan 523808, P.R. China.

**Keywords:** cytochalasin H (CyH), epithelial-mesenchymal transition (EMT), cancer stemness, YAP/TAZ, non-small cell lung cancer (NSCLC), mangrove

## Abstract

Our previous studies have isolated cytochalasin H (CyH) from endophytic fungus derived from mangrove and found that CyH induced apoptosis and inhibited migration and angiogenesis in non-small cell lung cancer (NSCLC) cells. In this study, we further investigated the effect of CyH on epithelial-mesenchymal transition (EMT) and cancer stemness of A549 and NCI-H460 NSCLC cells and the underlying mechanisms, especially the role of YAP/ TAZ signaling pathway in the process. Our results showed that CyH significantly inhibited invasive ability and the sphere formation of NSCLC cells. The expression of E-cadherin, an EMT epithelial marker, was obviously up-regulated, while the expression of Vimentin and N-cadherin, the EMT mesenchymal markers, was dramatically down-regulated by CyH treatment in NSCLC cells. Moreover, the expression of EMT-associated transcription factors including Slug, Twist1, and Snail1 and stemness markers including Nanog, Sox-2, and Oct-4 was significantly down-regulated by CyH treatment in NSCLC cells. Additionally, CyH significantly down-regulated YAP and TAZ expression and up-regulated LAST1/2 and MST1/2 expression, and CyH inhibited the interaction between YAP and TEAD. Furthermore, YAP knockdown abolished the effect of CyH on the expression of EMT- and stemness-related markers in NSCLC cells. Taken together, these results suggest that CyH inhibits EMT and cancer stemness of NSCLC cells *via* the regulation of YAP/TAZ signaling pathway.

## Introduction

Lung cancer is the leading cause of cancer-related mortality (18.4% of the total cancer-related mortality) in both sexes combined 1. Non-small cell lung cancer (NSCLC) comprises approximately 85% of lung cancer cases 2. NSCLC patients with the early-stage are treated by surgery, but recurrence and metastasis are very common at the stage I of NSCLC 2. Chemotherapeutic agents, such as gemcitabine, platinum compounds, and taxanes, can improve survival to a limited extent, but the overall survival rates of NSCLC patients remain low due to recurrence and metastasis 2,3.

Epithelial-mesenchymal transition (EMT) can promote recurrence and metastasis of NSCLC 4,5. EMT, the loss of epithelial phenotype and the gain of mesenchymal phenotype, is essential for the migratory and invasive capabilities of NSCLC 6,7. Most recently, Lafuente-Sanchis *et al.* found that the molecular EMT status of micrometastatic sentinel lymph nodes could be regarded as an independent prognosis predictor at the early stage of NSCLC patients 8. Moreover, Matsubara *et al.* also demonstrated that EMT was a poor prognosis predictor for the patients with stage IA lung adenocarcinoma, and EMT status might be considered as an indicator for administering adjuvant therapy 9. EMT can generate cells with properties of stem cells characterized by enhancing the expression of stemness-related markers 10. Therefore, the inhibitors of EMT and stemness may be promising drugs for the treatment of NSCLC.

Accumulating evidence demonstrates that Yes-associated protein (YAP)/transcriptional coactivator with PDZ-binding motif (TAZ) signaling pathway is involved in mediating EMT and cancer stemness 11-15. YAP and TAZ, two homologous transcriptional coactivators, are the final effectors of the Hippo signaling pathway 16. When the Hippo signaling pathway opens, one core kinase MST1/2 can phosphorylate and activate another core kinase LATS1/2. Then the activated LATS1/2 can phosphorylate YAP/TAZ and inactivate YAP/TAZ activity, inhibiting YAP/TAZ transfer to nucleus. Finally, YAP/TAZ in cytoplasm can bind to 14-3-3 or subject to proteasomal degradation 17,18. By contrast, when the Hippo signaling pathway closes, MSAT1/2 and LATS1/2 can be inactivated, leading to the inhibition of YAP/TAZ phosphorylation. Subsequently, the dephosphorylated YAP/TAZ can translocate to nucleus and bind to TEAD 17,18, enhancing TEAD-mediated transcription of the downstream genes and regulating the expression of the target genes such as EMT- and stemness-related genes. YAP/TAZ signaling pathway, activated in multiple human cancers, promotes cancer initiation, progression, and metastasis. Moreover, the increased expression or activation of YAP/TAZ is related to a poor prognosis 19,20. Specially, YAP/TAZ is a potential target for the therapy of squamous cell carcinoma including NSCLC 18,21. Therefore, the inhibition of YAP/TAZ is an important strategy for NSCLC therapy.

Mangroves grow in the salt-tolerant ecosystems with high biodiversity, and the abundance and diversity of mangrove resources provide the great potential for the discovery of new compounds 22. In recent years, multiple potential anticancer compounds have been found from mangrove-derived endophytic fungi 22-25. In our previous study, we have isolated cytochalasin H (CyH) from endophytic fungus *Phomopsis liquidambari* derived from mangrove, which could induce apoptosis and inhibit migration and angiogenesis in NSCLC cells both *in vitro* and *in vivo* 26,27. Tumor angiogenesis can cause EMT that generates cells with stemness properties 10. However, the effect of CyH on EMT and cancer stemness still remains unclear.

In this study, to the best of our knowledge, we found for the first time that CyH inhibited EMT and cancer stemness *via* the regulation of YAP/TAZ signaling pathway in A549 and NCI-H460 NSCLC cells. These findings indicate that CyH may be as a potential agent for the prevention and treatment of NSCLC.

## Materials and methods

### Drug and reagents

CyH (purity > 98% by HPLC) was extracted from the secondary metabolites of mangrove-derived endophytic fungus *Phomopsis liquidambari* by our lab 26. CyH was dissolved in 0.1% DMSO at 1 mM and diluted into different concentrations with culture medium. Recombinant human FGF-basic (bFGF) and recombinant human EGF were obtained from PeproTech Technology (New Jersey, CA, USA). Mouse anti-human YAP (1:1000 dilution; cat. no. 12395), and rabbit anti-human Phospho-YAP (p-YAP, Ser127, 1:1000 dilution; cat. no. 13008 ), LATS1 (1:1000 dilution; cat. no. 3477), LATS2 (1:1000 dilution; cat. no. 5888), MST1 (1:1000 dilution; cat. no. 3682), MST2 (1:1000 dilution; cat. no. 3952), TAZ (1:1000 dilution; cat. no. 3418), N-Cadherin (1:1000 dilution; cat. no. 13116), Vimentin (1:1000 dilution; cat. no. 5741), Snail1 (1:1000 dilution; cat. no. 3879), Slug (1:1000 dilution; cat. no. 9585), Twist1 (1:1000 dilution; cat. no. 46702), Sox2 (1:1000 dilution; cat. no. 14962), Oct-4A (1:1000 dilution; cat. no. 2750), Nanog (1:1000 dilution; cat. no. 4903), and TEAD (1:1000 dilution; cat. no. 12293) primary antibodies were purchased from Cell Signaling Technology (Danvers, MA, USA). Mouse anti-human E-Cadherin antibody (1:1000 dilution; cat. no. 4903) was purchased from Millipore Technology (Boston, MA, USA). Goat-anti rabbit-HRP (1:2000 dilution; cat. no. 7074S) and goat-anti mouse-HRP (1:2000 dilution; cat. no. 7076S) secondary antibodies and rabbit anti-human IgG (1:1000 dilution; cat. no. 4096) were obtained from Cell Signaling Technology (Danvers, MA, USA). One Step SYBR^®^ PrimeScript^®^ RT-qPCR kit (No. DRR086A) was from TaKaRa Biotechnology Co., Ltd. (Dalian, China). BD Matrigel^TM^ Basement Membrane Matrix High Concentration (No. 354248) was from BD Biosciences (Bedford, MA, USA). Protein A Magnetic Beads were from Cell Signaling Technology (Danvers, MA, USA). Lipofectamine 3000 transfection reagent was from Invitrogen (Carlsbad, CA, USA).

### Cell lines and cell culture

Human A549 and NCI-H460 NSCLC cell lines were purchased from ATCC (Rockville, MD, USA) and the Chinese Academy of Sciences Cell Bank of Type Culture Collection (Shanghai, China), respectively. A549 and NCI-H460 cells were grown in RPMI-1640 media containing 10% fetal bovine serum (FBS) in a 5% CO_2_ incubator at 37 °C. All cells used in this study were in the logarithmic phase of growth.

### Invasion assay

The transwell chambers with 8 μm pore (Corning, NY, USA) were pre-coated with Matrigel that was diluted with serum-free medium at 1:8 ratio. A549 and NCI-H460 cells (8.0 × 10^5^) were pretreated with CyH (0, 0.05, 0.1, 0.2, 0.4, and 0.8 μM) for 16 h. Afterwards, the treated cells were washed three times with PBS and then the washed cells were plated on the top of each chamber, and the medium containing 20% FBS was put in the lower chamber as a chemoattractant. 36 h incubation later, the cells that did not pass through the filter were removed by a cotton swab and the cells that pass through the filter were stained with 0.1% crystal violet. The invasive cells were counted at least 5 randomized fields under a microscope and the average invasive cell number per field was calculated.

### Sphere formation assays

A549 and NCI-H460 cells were treated with CyH at 1.51 μM for 16 h. Afterwards, the treated cells were washed three times with PBS, and then the washed cells (5000/well) were seeded into 6-well ultra-low attachment cluster plates (Corning, NY, USA) and grown in serum-free DMEM/F12 medium (Invitrogen, Carlsbad, CA, USA) containing 20 ng/ml bFGF, 20 ng/ml EGF, 2% B27, 0.4% BSA, and 5 μg/ml insulin. Two weeks later, the formation of spheres was photographed and the number of the spheres was counted.

### YAP- siRNA transfection

One day before transfection, the cells were seeded into a 6-well plate at 50% confluence without antibiotics. The YAP-siRNA (5′-GCAUCUUCGACAGUCUUC- UTT-3′) was synthesized by Sangon Biotech (Shanghai, China). Non-specific siRNA (NS-siRNA) was used as a control. The siRNA transfection was performed using Lipofectamine 3000 according to the manufacturer's instructions.

### Western blotting

The method was as described previously 26,27. Briefly, A549 and NCI-H460 cells were respectively treated with different concentrations (0, 1.51, 3.13, 6.25, 12.5, and 25 μM) of CyH for 16 h. Afterwards, the cells were lysed on ice with RIPA lysis buffer (Beyotime Institute of Biotechnology, Shanghai, China) containing protease inhibitors for 1 h. The lysates were ultra-sonicated and centrifuged at 12,000 rpm for 10 min. Protein concentration was detected by the BCA protein assay. The protein samples (100 μg) were run on 10% SDS-PAGE and transferred to PVDF membranes. After being blocked with 5% non-fat milk or BSA for more than 2 h at room temperature, the membranes were respectively incubated overnight at 4 °C with specific primary antibodies against E-cadherin, N-cadherin, Vimentin, OCT-4, SOX-2, Nanog, YAP, p-YAP, TAZ, LATS1/2, and MST1/2 (1:1000), followed by incubation for 1 h with HRP-conjugated secondary antibodies (1:2000). The signals were examined by ECL reagents and the density was analyzed by ImageJ software 26,27.

### RT-qPCR

The method was as described previously 27,28. Briefly, A549 and NCI-H460 cells were respectively treated with CyH (0, 1.51, 3.13, 6.25, 12.5, and 25 μM) for 16 h. Total RNA in the treated and untreated cells was extracted using Trizol reagent and the mRNA levels of the target genes were analyzed using One Step SYBR^®^ PrimeScript^®^ RT-PCR (TaKaRa, China). All primers were synthesized by Sangon Biotech (Shanghai, China) and the sequences were listed in Table 1. The reaction conditions were as follows: 42 °C for 5 min, then 95 °C for 10 s, followed by 40 cycles (95 °C for 5 s and 60 °C for 31 s). *β-actin* was used as a control to normalize the relative mRNA levels of all target genes.

### Co-Immunoprecipitation (Co-IP) assay

A549 and NCI-H460 cells were treated with CyH at 3.13 μM for 16 h. Then, the cells were collected and washed three times with PBS. Afterwards, the washed cells were seeded into six-well plates and incubated with the ice-cold lysis buffer (200~400 μL/well) on ice for 30 min with periodic mixing. Afterwards, the cell lysates were transferred to a microcentrifuge tube and centrifuged at 4 °C (14,000 g for 15 min). The supernatant was incubated with anti-YAP antibody overnight at 4 °C, and the incubation with rabbit anti-human IgG served as a negative control. Protein A magnetic beads were added into the mixture and incubated with agitation for 2 h at room temperature. After centrifugation for 30 s at 14,000 g, the beads (magnetic bead-Ab-Ag complex) were collected and washed three times with PBS for 10 min each time. The precipitants were dissolved with the SDS loading buffer for the analysis of YAP and TEAD protein expression by western blotting as described above.

### Statistical analysis

All experiments were repeated more than 3 times. All data were shown as 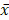
± *s*. One-way ANOVA assay in SPSS 16.0 statistical software was used to analyze data. *P*<0.05 indicated the statistical significance.

## Results

### CyH inhibited invasive ability of NSCLC

**Figure 1:**
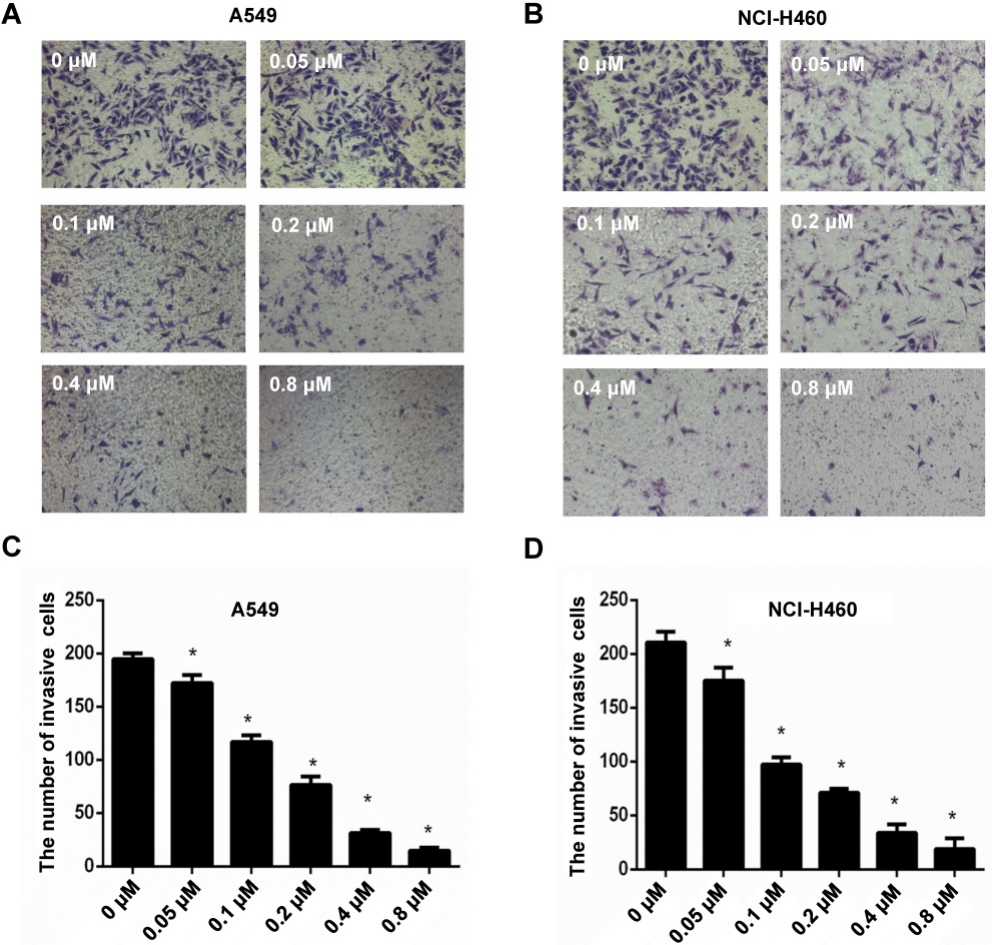
Effect of CyH on the invasive ability of NSCLC cells. NSCLC cells (A549 and NCI-H460) were plated on the top chambers (Matrigel in the upper chamber) and respectively treated with different concentrations (0, 0.05, 0.10, 0.20, 0.4 and 0.8 µM) of CyH for 36 h. Afterwards, the number of invasive cells was counted under a microscope. (**A,B**) The representative results of three independent experiments (A:A549 cells, B:NCI-H460 cells; magnification, ×200). (**C,D**) The number of invasive cells. Compared with the control (0 µM), *P<0.05.

To explore the effect of CyH on invasive ability of NSCLC cells, A549 and NCI-H460 NSCLC cells were respectively treated with 0, 0.05, 0.1, 0.2, 0.4 and 0.8 μM of CyH for 16 h. Our results showed that CyH inhibited invasion in A549 and NCI-H460 cells (**Figure 1A and B**), and the number of invasive cells was obviously decreased with the increase of CyH concentration (*P*<0.05, **Figure 1C and D**), indicating that CyH dose-dependently inhibited the invasive ability in NSCLC cells.

### CyH inhibited EMT of NSCLC cells

**Figure 2:**
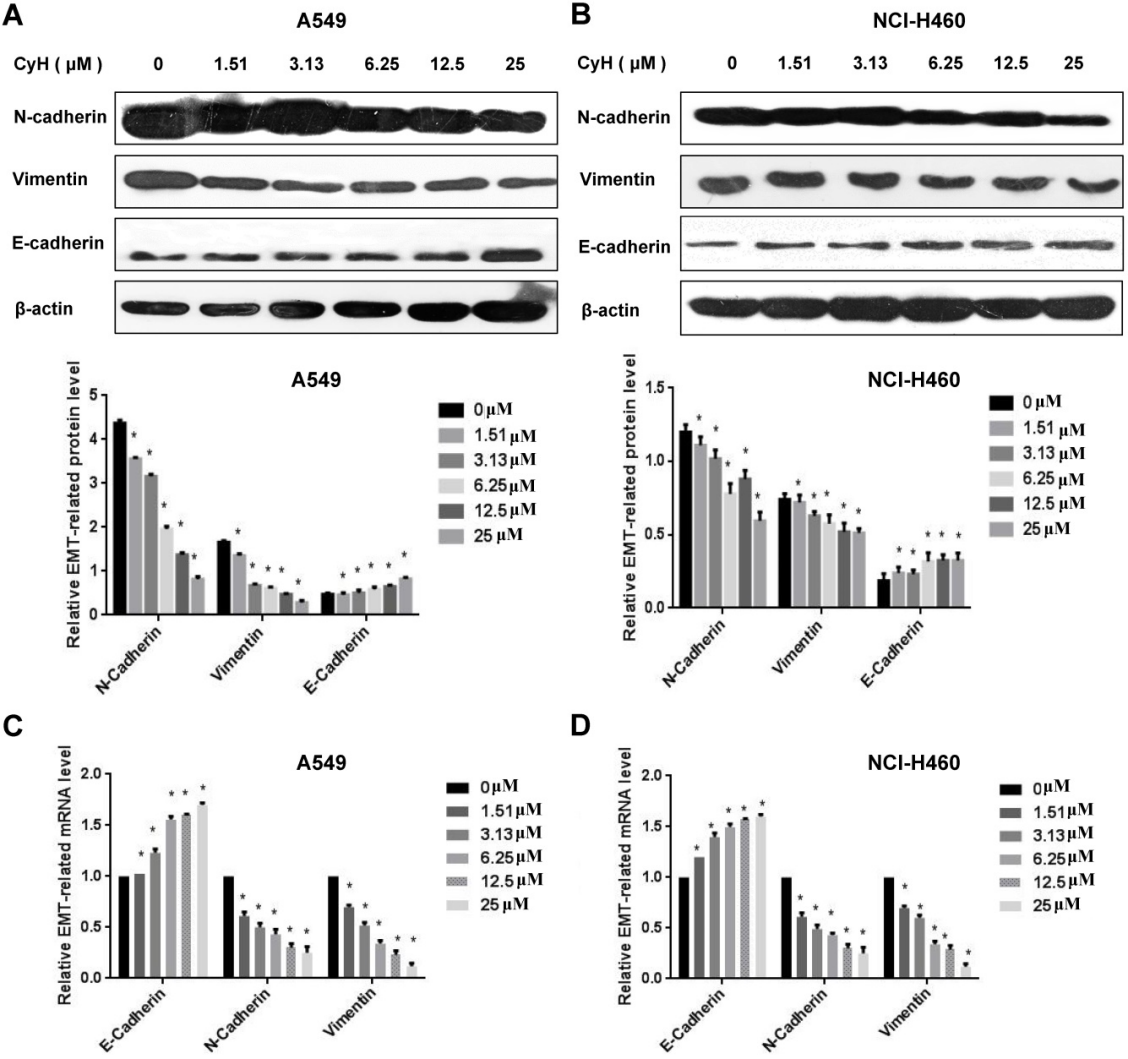
Effect of CyH on the expression of EMT-related markers in NSCLC cells. NSCLC cells (A549 and NCI-H460) were respectively treated with different concentrations (0, 1.51, 3.13, 6.25, 12.5 and 25 µM) of CyH for 16 h, followed by the analysis of EMT-related marker expression. (**A,B**) N-Cadherin, Vimentin, and E-Cadherin protein levels were analyzed by western blotting (A:A549 cells, B:NCI-H460 cells; up: the representative results of three independent experiments, down: the results of density for three independent experiments). (**C,D**) N-Cadherin, Vimentin, and E-Cadherin mRNA levels were determined by RT-qPCR. The results represented the mean ± SD from three replicate experiments. Compared with control (0 µM), ^*^P<0.05.

**Figure 3:**
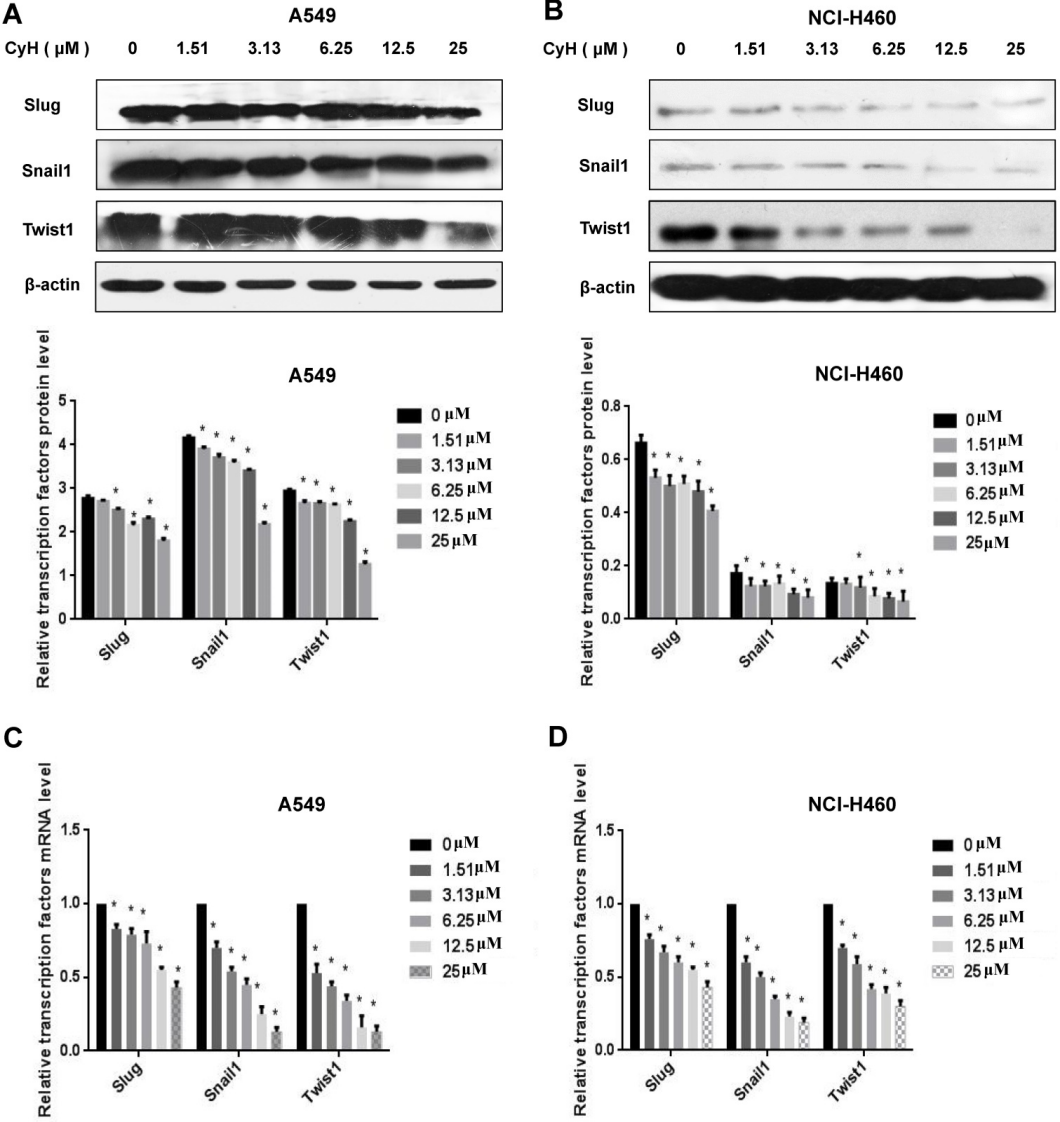
Effect of CyH on the expression of EMT-related transcription factors in NSCLC cells. NSCLC cells (A549 and NCI-H460) were respectively treated with different concentrations (0, 1.51, 3.13, 6.25, 12.5 and 25 µM) of CyH for 16 h, followed by the analysis of EMT-related transcription factor expression. (**A,B**) Snail1, Slug, and Twist1 protein levels were analyzed by western blotting (A:A549 cells, B:NCI-H460 cells; up: the representative results of three independent experiments, down: the results of density for three independent experiments). (**C,D**) Snail1, Slug, Twist1 mRNA levels were determined by RT-qPCR. The results represented the mean ± SD from three replicate experiments. Compared with control (0 µM), ^*^P<0.05.

EMT is an important step of migration and invasion. In our previous study 26 and the present study, we have found that CyH inhibited migration and invasion in A549 and NCI-H460 NSCLC cells. To further explore the effect of CyH on EMT, A549 and NCI-H460 cells were respectively treated with 0, 1.51, 3.13, 6.25, 12.5, and 25 μM of CyH for 16 h, followed by the analysis of expression of EMT-associated markers. Our results demonstrated that CyH promoted the expression of EMT epithelial marker E-Cadherin (**Figure 2A-D**, *P*<0.05), and inhibited the expression of EMT mesenchymal markers (N-Cadherin and Vimentin) at both protein and mRNA levels in A549 and NCI-H460 cells (**Figure 2A-D**, *P*<0.05). Furthermore, we further found that CyH dramatically suppressed the expression of EMT transcription factors (Snail1, Slug, and Twist1) in A549 and NCI-H460 cells (*P*<0.05, **Figure 3**). Taken together, these results suggested that CyH inhibited EMT of A549 and NCI-H460 cells.

### CyH inhibited the sphere formation and the expression of stemness-related markers in NSCLC cells

**Figure 4:**
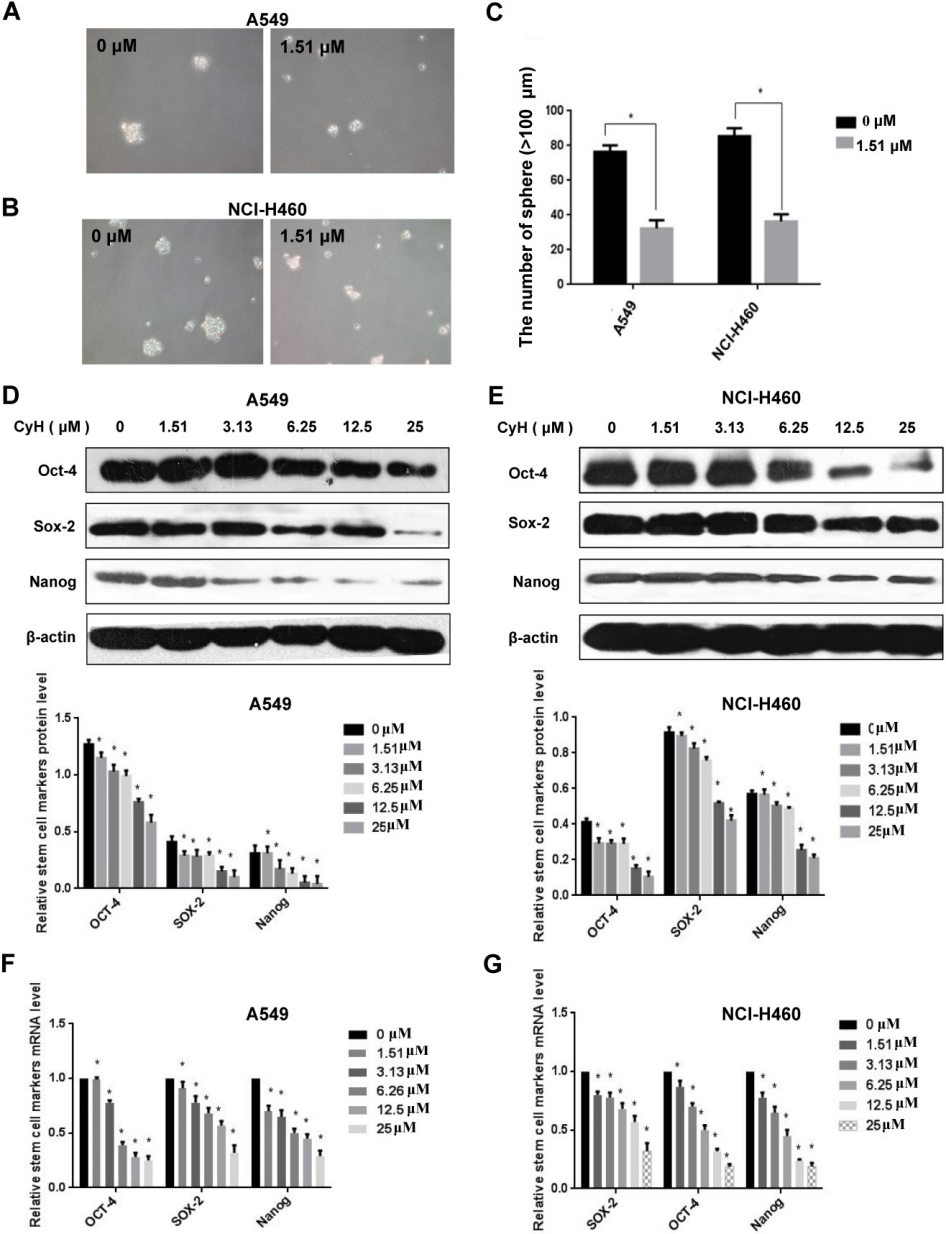
Effect of CyH on the expression of stemness-related markers in NSCLC cells. (**A-C**) NSCLC cells were treated with CyH at 1.51 µM for 10 days. Afterwards, the stem-like sphere formation was observed and the number of spheres was counted under fluorescence microscope (magnification, ×200). The results are representative of three independent experiments (A:549, B:NCI-H460). The number of stem-like spheres (C). (**D,E**) NSCLC cells were respectively treated with different concentrations (0, 1.51, 3.13, 6.25, 12.5 and 25 µM) of CyH for 16 h. Afterwards, OCT-4, SOX-2, and Nanog protein levels were analyzed by western blotting (D:A549 cells, E:NCI-H460 cells; up: the representative results of three independent experiments, down: the results of density for three independent experiments). (**F,G**) OCT-4, SOX-2, and Nanog mRNA levels were determined by RT-qPCR. All results represented mean ± SD from three replicate experiments. Compared with control (0 µM), ^*^P<0.05.

EMT can promote cells with properties of stemness 10, and the sphere formation is one of the important characteristics of stem cells. To further study the effect of CyH on NSCLC stemness, A549 and NCI-H460 cells were treated with CyH at 1.51 μM for 10 days, followed by the analysis of the sphere formation. Our results showed that CyH inhibited the sphere formation of A549 and NCI-H460 cells (**Figure 4A and B**). Moreover, the number of sphere formation (>100 μm) was significantly reduced by CyH treatment (*P*<0.05, **Figure 4C**) in A549 and NCI-H460 cells. Furthermore, we further studied the effect of CyH on the expression of stemness-related markers (Sox-2, Oct-4, and Nanog). As expected, CyH dramatically inhibited Sox-2, Oct-4, and Nanog expression at both protein (**Figure 4D and E**) and mRNA (**Figure 4F and G**) levels (*P*<0.05). Collectively, CyH suppressed the stemness of A549 and NCI-H460 cells.

### CyH inhibited EMT through the suppression of YAP/TAZ signaling pathway in NSCLC cells

**Figure 5:**
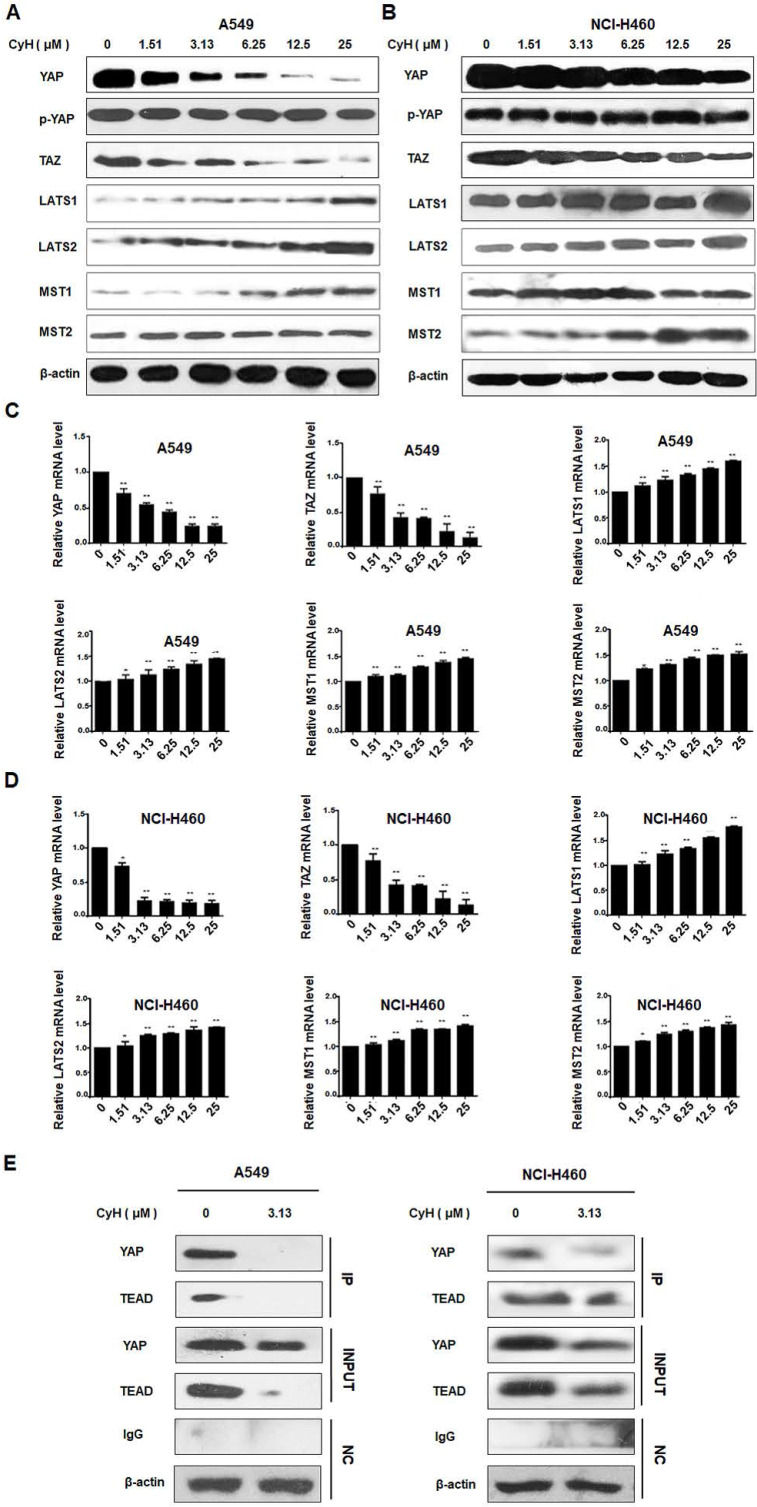
Effect of CyH on the activation of YAP/TAZ signaling pathway in NSCLC cells. NSCLC cells (A549 and NCI-H460) were respectively treated with different concentrations (0, 1.51, 3.13, 6.25, 12.5 and 25 µM) of CyH for 16 h, followed by the analysis of expression of YAP/TAZ signaling-related factors. (**A,B**) YAP, p-YAP, TAZ, LATS1/2, and MST1/2 protein levels were analyzed by western blotting (A:A549 cells, B:NCI-H460 cells). (**C,D**) YAP, p-YAP, TAZ, LATS1/2, and MST1/2 mRNA levels were determined by RT-qPCR. The results represented mean ± SD from three replicate experiments. Compared with control (0 µM), ^*^P<0.05, ^**^P<0.01. (**E**) The interaction between YAP and TEAD was detected by co-immunoprecipitation.

YAP/TAZ signaling pathway can mediate EMT and cancer stemness 11-15. To explore whether CyH inhibited EMT by suppressing YAP/TAZ signaling pathway, we analyzed YAP, p-YAP, TAZ, LATS1/2, and MST1/2 protein expression by Western blotting. As shown in **Figure 5A and B**, YAP and TAZ protein expression was down-regulated and LATS1/2 and MST1/2 protein expression was up-regulated in CyH-treated A549 and NCI-H460 cells. However, CyH had no obvious effect on the phosphorylation level of YAP in both A549 and NCI-H460 cells. At the same time, LATS1/2, MST1/2, YAP, and TAZ mRNA levels were analyzed by RT-qPCR. As shown in **Figure 5C and D**, CyH significantly down-regulated YAP and TAZ mRNA levels, while CyH remarkably up-regulated LATS1/2 and MST1/2 mRNA levels in A549 and NCI-H460 cells (*P*<0.05). Moreover, CyH inhibited the expression of YAP and TEAD proteins and the interaction between the two proteins in A549 and NCI-H460 cells (**Figure 5E**). Collectively, these results suggested that CyH inhibited the activation of YAP/TAZ signaling pathway in NSCLC cells.

**Figure 6:**
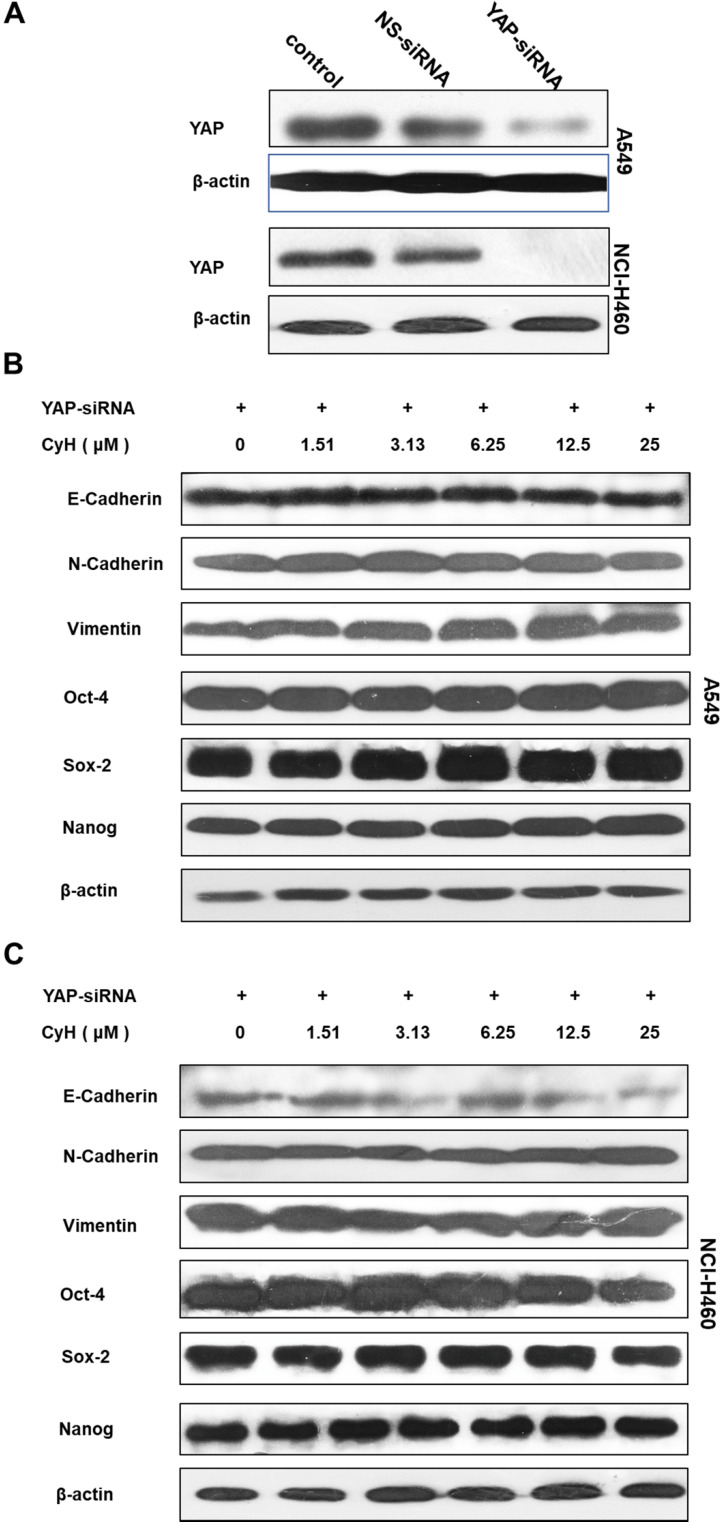
Role of YAP in the effect of CyH on EMT- and stemness-related protein expression. (**A-C**) NSCLC cells were transiently transfected with YAP-siRNA and respectively treated with different concentrations (0, 1.51, 3.13, 6.25, 12.5 and 25 µM) of CyH for 16 h, followed by Western blotting. The expression of YAP (A) and the expression of E-cadherin, N-cadherin, Vimentin, OCT-4, SOX-2, and Nanog (B:A549, C:NCI-H460)

To further validate the role of YAP/TAZ signaling pathway in the effect of CyH on EMT and cancer stemness, A549 and NCI-H460 cells were transiently transfected with YAP-siRNA, followed by the analysis for EMT-related protein expression. Our results showed that YAP-siRNA obviously knocked down the expression of YAP (**Figure 6A**). As expected, the knockdown of YAP inhibited the effect of CyH on the expression of EMT-related proteins including N-Cadherin, Vimentin, and E-Cadherin and stemness-related proteins including OCT-4, SOX-2, and Nanog (**Figure 6B and C**).

## Discussion

Cytochalasins are a sort of microfilament-directed agents 28. Recently, a growing body of evidence has demonstrated that cytochalasins also exhibit potential anticancer activities both *in vitro* and *in vivo* 29-32. In our previous studies, we isolated CyH, one kind of cytochalasins, from endophytic fungus *Phomopsis sp*. derived from mangrove, and found that CyH could induce apoptosis and inhibit migration in A549 NSCLC cells 26. In the present study, we further found that CyH dose-dependently inhibited invasion in A549 and NCI-H460 NSCLC cells (**Figure 1**). In our previous studies, we demonstrated that CyH suppressed NSCLC angiogenesis by inhibiting hypoxia inducible factor (HIF)-1α protein accumulation and vascular endothelial growth factor (VEGF) expression 27. EMT is a key step of migration and invasion. Moreover, HIFs were considered as the master regulators of stemness properties, and VEGF-mediated angiogenesis was reported to be associated with EMT-induced cancer stemness 33-35. Therefore, in the present study, we further investigated the effect of CyH on EMT and cancer stemness of NSCLC. Our results showed that CyH inhibited EMT (**Figures 2 and 3**). Additionally, we also found that CyH significantly down-regulated the expression of Oct-4, Sox-2, and Nanog, the stemness-related markers (**Figure 4D-G**). Furthermore, we demonstrated that CyH at 1.51 μM significantly inhibited sphere formation (**Figure 4A-C**). Collectively, our results indicate that CyH suppresses EMT and cancer stemness in NSCLC cells, suggesting that CyH may be a potential chemotherapeutic agent in NSCLC therapy.

YAP/TAZ signaling pathway can regulate EMT and cancer stemness. YAP was found to regulate the EMT-associated genes by inducing SOX-2 expression in cooperation with Oct-4 in NSCLC cells 12 and to directly up-regulate SOX-9 and induce stem-like properties in esophageal cancer cells 13. TAZ was demonstrated to promote cancer stem cell (CSC) traits in breast cancer cells 14, and overexpression of TAZ in mammary epithelial cells was reported to induce EMT 15. Moreover, TAZ was found to be essential for metastasis and chemo-resistance of breast cancer stem cells 36. These reports indicate that YAP/TAZ signaling pathway can promote tumor initiation, progression, and metastasis by mediating EMT and cancer stemness. Interestingly, YAP/TAZ was regarded as an important target for the prevention and treatment of NSCLC 18,21. Therefore, in the present study, we further explore the effect of CyH on the YAP/TAZ signaling pathway. Our results showed that CyH dramatically inhibited YAP and TAZ expression but had no obvious effect on YAP phosphorylation, indicating that CyH down-regulated dephosphorylated YAP and TAZ levels. MST1/2 and LATS1/2, the upstream core kinases of YAP/TAZ, are tumor inhibitors that can suppress the oncogenic nuclear function of YAP/TAZ and TEAD 37. Here, we further analyze the effect of CyH on MST1/2 and LATS1/2 expression. Our results showed that CyH dramatically promoted the expression of MST1, MST2, LATS1, and LATS2 (**Figure 5A-D,** *P*<0.05). YAP-TEAD interaction can promote tumor growth and metastasis 17, and YAP/TAZ-TEAD transcriptional complex has been demonstrated to be a potential target for cancer therapy 38-40. So, we further analyze the effect of CyH on the interaction between YAP and TEAD in this study. As expected, in the present study, we demonstrated that the interaction between YAP and TEAD was significantly attenuated by CyH treatment (**Figure 5E**). Collectively, these results suggest that CyH can inhibit the activation of YAP/TAZ signaling pathway.

To further verify whether CyH inhibited EMT and cancer stemness in NSCLC cells *via* the YAP/TAZ signaling pathway, YAP-siRNA was transfected into NSCLC cells. Our results showed that YAP knockdown abrogated the effect of CyH on the expression of EMT- and cancer stemness-related markers (**Figure 6**), suggesting that YAP/TAZ pathway may be involved in the effect of CyH on EMT and cancer stemness in NSCLC cells.

In summary, in the present study, we firstly demonstrated that CyH inhibited EMT and cancer stemness in A549 and NCI-H460 cells. Moreover, YAP/TAZ signaling pathway was involved in mediating this process. These results indicate that CyH may be a potential chemotherapeutic agent for NSCLC and provide a novel molecular mechanism for the role of CyH in the prevention and treatment of NSCLC.

## Figures and Tables

**Table 1:** Primer sequences

Name	Sequences (5′→ 3′)	GenBank No.
TAZ	Forward: CTCCCACTTCAGCTTGG	NM_000116.5
	Reverse: TCTGGTAGACGCCATCTCCT	
YAP	Forward: TAGCCCTGCGATGCCAGTTA	NM_001130145.3
	Reverse: TCATGCTTAGTCCACTGTCTGT	
Twist1	Forward: GGAGTCCGCAGTCTTACGAG	NM_000474.4
	Reverse: CCAGCTTGAGGGTCTGAATC	
Slug	Forward: GAGCATACAGCCCCATCACT	NM_003068.5
	Reverse: GGGTCTGAAAGCTTGGACTG	
SOX-2	Forward: TACAGCATGTCCTACTCGCAG	NM_021783.5
	Reverse: GAGGAAGAGGTAACCACA	
OCT-4	Forward: CTTGAATCCCGAATGGAAAGG	NM_001285987.1
	Reverse: GTGTATATCCCAGGGTGATC	
E-Cadherin	Forward: TTGCTACTGGAACAGGGACAC	NM_004360.5
	Reverse: CCCGTGTGTTAGTTCTGCTGT	
N-Cadherin	Forward: TTATCCTTGTGCTGATGTTT	NM_001792.5
	Reverse: TCTTCTTCTCCTCCACCTTC	
Snail1	Forward: TCCTTCGTCCTTCTCCTCTACTT	NM_005985.4
	Reverse: TGTTGCAGTATTTGCAGTTG	
Vimenin	Forward: GAGAACTTTGCCGTTGAAGC	NM_003380.5
	Reverse: TCCAGCAGCTTCCTGTAG	
Nanog	Forward: ACCTTCCAATGTGGAGCAAC	NM_024865.4
	Reverse: GAATTTGGCTGGAACTGCAT	
LATS1	Forward: GTCCTTCGTGTGGGCTACAT	NM_004690.4
	Reverse: CGAGGATCTTCGGTTGACAT	
LATS2	Forward: CGCCATACGCCTTTAAGTTC	NM_014572.3
	Reverse: TGGCCCTCTTTAACCTGTTG	
MST1	Forward: TTCACGTTTACCTCCGAACC	XM_011533738.3
	Reverse: TGCCACACTTCTCAAACTGC	
MST2	Forward: CATGAGGAACAGCAACGAGA	NM_006281.4
	Reverse: TATCACCATGGTCCCCAAGT	
β-actin	Forward: TGACGTGGACATCCGCAAAG	NM_001101.5
	Reverse: CTGGAAGGTGGACAGCGAGG	
