# Cytochalasin H isolated from mangrove-derived endophytic fungus inhibits epithelial-mesenchymal transition and cancer stemness *via* YAP/TAZ signaling pathway in non-small cell lung cancer cells: Erratum

**DOI:** 10.7150/jca.86288

**Published:** 2023-05-30

**Authors:** Zihan Xiu, Jiao Liu, Xin Wu, Xiangyong Li, Sanzhong Li, Xiaofeng Wu, Xiaohua Lv, Hua Ye, Xudong Tang

**Affiliations:** 1 Collaborative innovation center for antitumor active substance research and development, Institute of Biochemistry and Molecular Biology, Guangdong Medical University, Zhanjiang 524023, P.R. China.; 2 Guangdong Key Laboratory for Research and Development of Natural Drugs, Marine Medical Research Institute of Guangdong Zhanjiang, Department of Pharmacology, Guangdong Medical University, Zhanjiang 524023, P.R. China.; 3 Southern Marine Science and Engineering Guangdong Laboratory (Zhanjiang), Zhanjiang 524023, P.R. China.; 4 Guangdong Provincial Key Laboratory of Medical Molecular Diagnostics, Dongguan Key Laboratory of Medical Bioactive Molecular Developmental and Translational Research, Guangdong Medical University, Dongguan 523808, P.R. China.

In the original version of our article, there were the inadvertent errors in Figure 6. Specifically, the result of β-actin expression of NCI-H460 cells in Figure 6A was wrongly pasted. Additionally, the result of Nanog expression in Figure 6C showed one more band because one more concentration (50 µM) was done. The correct Figure 6 is provided below. This correction will not affect the results and conclusions. The authors apologize for any inconvenience the errors may have caused.

## Figures and Tables

**Figure 6:**
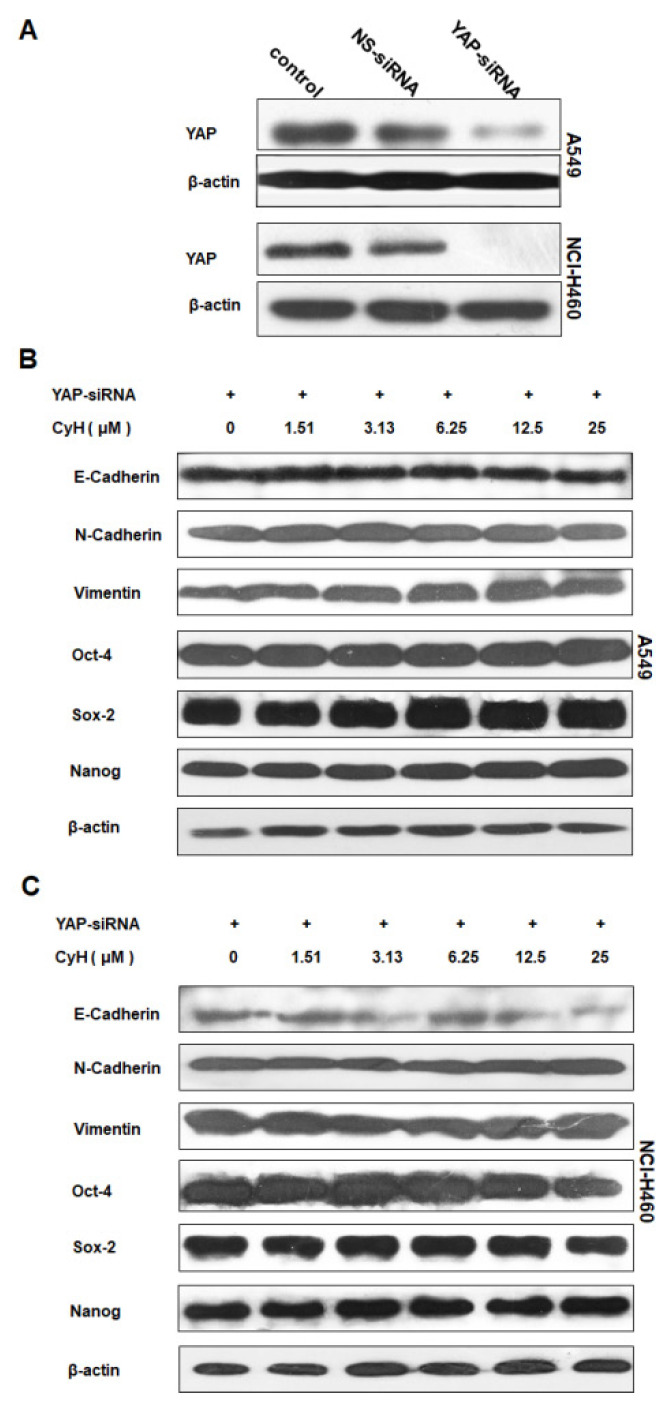
Role of YAP in the effect of CyH on EMT- and stemness-related protein expression. (A-C) NSCLC cells were transiently transfected with YAP-siRNA and respectively treated with different concentrations (0, 1.51, 3.13, 6.25, 12.5 and 25 µM) of CyH for 16 h, followed by Western blotting. The expression of YAP (A) and the expression of E-cadherin, N-cadherin, Vimentin, OCT-4, SOX-2, and Nanog (B:A549, C:NCI-H460).

